# Androgen receptor mitigates postoperative disease progression of hepatocellular carcinoma by suppressing CD90+ populations and cell migration and by promoting anoikis in circulating tumor cells

**DOI:** 10.18632/oncotarget.10186

**Published:** 2016-06-20

**Authors:** Hsueh-Chou Lai, Chun-Chieh Yeh, Long-Bin Jeng, Shang-Fen Huang, Pei-Ying Liao, Fu-Ju Lei, Wei-Chun Cheng, Cheng-Lung Hsu, Xiujun Cai, Chawnshang Chang, Wen-Lung Ma

**Affiliations:** ^1^ Graduate Institution of Clinical Medical Science, and Graduate Institution of Cancer Biology, China Medical University, Taichung 40403, Taiwan; ^2^ Sex Hormone Research Center, Organ Transplantation Center, Research Center for Tumor Medical Science, and Department of Gastroenterology, China Medical University/Hospital, Taichung 40403, Taiwan; ^3^ Division of Hematology-Oncology, Department of Internal Medicine, Chang Gung University/Memorial Hospital, Taoyuan 333, Taiwan; ^4^ Chawnshang Chang Liver Cancer Center, Department of General Surgery, Sir Run-Run Shaw Hospital, Zhejiang University, Hangzhou 310016, China; ^5^ George Whipple Laboratory for Cancer Research, Department of Pathology, The Wilmot Cancer Center, University of Rochester Medical Center, Rochester, NY 14623, USA

**Keywords:** AR, HCC recurrence, CTC, CD90, anoikis

## Abstract

**Purpose:**

Although hepatectomy and liver transplantation surgery for hepatocellular carcinoma (HCC) are effective treatment modalities, the risk of recurrence remains high, particularly in patients with a high number of circulating tumor cells (CTCs) expressing cancer stem/progenitor cell markers. Androgen receptor (AR) signaling has been shown to suppress HCC metastasis in rodent models of HCC. In this study, we investigated whether AR is associated with postoperative HCC recurrence.

**Experimental Design:**

CTCs were obtained from patients with HCC who had undergone hepatectomy to investigate whether they are associated with disease outcome. AR knockout was introduced in two mouse models of spontaneous HCC (carcinogen- and hepatitis B virus-related HCC) to delineate the role that AR plays in HCC recurrence. Biological systems analysis was used to investigate the cellular and molecular mechanisms.

**Results:**

We found that the expression of AR in CTCs was negatively associated with HCC recurrence/progression after hepatectomy. Our results suggest that AR-mediated suppression of HCC recurrence/progression is governed by a three-pronged mechanism. First, AR suppresses the expression of CD90 in CTCs by upregulating Histone 3H2A. Second, AR suppresses cell migration at the transcriptome level. Third, AR promotes anoikis of CTCs via dysregulation of cytoskeletal adsorption.

**Conclusions:**

The results indicate that AR expression may be the gatekeeper of postoperative HCC recurrence. Therefore, targeting AR in presurgical down-staging procedures may serve as a secondary prevention measure against HCC recurrence in the future.

## INTRODUCTION

Hepatocellular carcinoma (HCC) is one of the most prevalent types of liver cancer worldwide [1, 2]. The androgen receptor (AR) has been demonstrated to be associated with liver carcinogenesis in mouse models [3, 4] and in humans [5]. Studies have shown that high serum testosterone levels and a low number of AR-CAG repeats are associated with an increased risk of hepatitis B virus (HBV)-related HCC [6], indicating that androgen/AR signaling contributes to the higher prevalence of HCC in men. Numerous animal studies have revealed that AR acts as a promoter of carcinogenesis in the liver [3, 4, 7]. However, clinical trials have demonstrated that anti-androgenic treatment does not result in a survival benefit [8, 9]. Therefore, many researchers have started studying about the role that AR plays not only in the early phase of cancer development but also in the progression, metastasis, and recurrence of liver cancer. Animal studies have demonstrated that AR acts as a suppressor of cancer progression by inhibiting cancer cell invasion [10] and by promoting cell detachment-induced apoptosis (anoikis) [11]. However, whether the level of AR expression plays a role in suppressing HCC recurrence has yet to be evaluated.

Although curative hepatectomy and liver transplantation surgery are effective treatments for HCC [12], the risk of recurrence remains high with reported 3-year recurrence rates ranging from 40% to 70% after hepatectomy [13] and 20%–50% after living donor liver transplantation surgery [14]. Possible reasons for the high rates of recurrence after surgery include primary cancer cell dissemination, the survival of extravasated cancer cells (circulating tumor cells; CTCs) [15], the colonization capacity of CTCs [16], the number of CTCs expressing the membrane protein Thy-1 (CD90), a cancer stem/progenitor cell (CSPC) marker gene [17], and cancer cell mobility [18]. However, the regulatory mechanisms governing the process of recurrence are still unclear.

In this study, we found that AR expression was associated with a reduction in primary tumor CD90+ populations, a reduction in cancer cell migration, and an increase in CTC death, indicating that increased expression of AR might protect against postoperative HCC recurrence.

## RESULTS

### AR and CD90+ expression are inversely correlated in primary HCC

**Figure 1:**
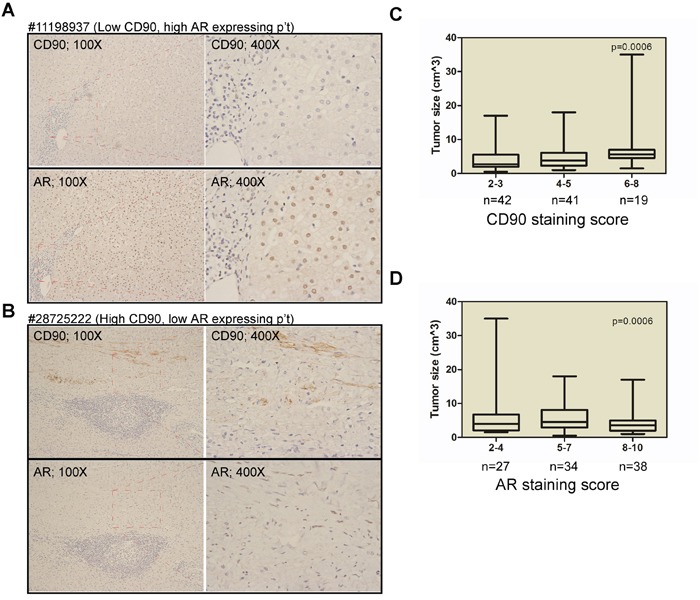
Inversed AR and CD90 expression patterns in HCC specimen **A.** Immunohistochemistry (IHC) staining for AR and CD90 in serial tumor sections from a representative patient (#11198937) revealed a low CD90 but high AR expression pattern. **B.** IHC staining for AR and CD90 in serial tumor sections from a representative patient (#28725222) revealed a high CD90 but low AR expression pattern. **C.** The association between CD90 expression and tumor size in HCC patients. Weak staining (IHC score 2~3) was noted in samples from 42 patients, moderate staining (IHC score 4~5) was observed in samples from 41 patients, and strong staining (IHC score 6~8) was noted in samples from 19 patients. **D.** The association between AR expression and tumor size. Weak staining (IHC score 2~4) was noted in samples from 27 patients, moderate staining (IHC score 5-7) was observed in samples from 34 patients, and strong staining (IHC score 8-10) was noted in samples from 38 patients.

In order to examine the role of AR expression in hepatic surgery HCC patients, in terms of its association with disease progression and the recurrence, we performed a single-cohort study as described in the Materials and Methods section; the demographic data are presented in Table 1. We found that the AR staining scores were not associated with sex, HBV or hepatitis C virus (HCV) infection, or serum alpha-fetoprotein (AFP) levels. Neither AR staining score were associated with TNM stage or disease-free survival in the study cohort. However, the high AR staining scores was associate smaller tumor size. These findings are consistent with those reported by Soong [19] and Boix [20] et al. We then examined AR and CD90 staining score in the primary tumor using serial sections. We found that AR and CD90 expression are inversely expression. As shown in Figure 1A and 1B, low CD90 expressing lesions (patient #11198937) have high AR expression. Conversely, high CD90 expressing lesions (patient #28725222) have low AR expression. Regarding the association between AR and CD90 expression and the disease status, we found that a higher CD90 staining score (score 6~8) is associated with larger tumor size (Figure 1C). Furthermore, higher AR expression (score 8~10) is inversely associated with smaller tumor size (Figure 1D).

**Table 1:** Characteristics of the HCC patients associated with AR staining score in immunohistochemistry

	AR staining score			
	n	mean	SD	p-value
All	90	5.86	1.98	-
Sex				0.2073
Female	20	6.35	2.01	
Male	70	5.71	1.96	
HBV				0.1849
Negative	45	5.58	1.98	
Positive	45	6.13	1.96	
HCV				0.6187
Negative	59	5.78	1.98	
Positive	31	6.0	2.0	
AFP (pre-OP)				0.2916
<20ng/ml	49	5.65	1.95	
≥20ng/ml	41	6.1	2.01	
TNM stage				0.9453
<II	32	5.88	2.12	
≥II	58	5.85	1.92	
Tumor size				
<5 cm	56	6.18	2.03	0.0465
≥5 cm	34	5.32	1.8	
Disease-free survival				0.9773
Yes	64	5.86	1.97	
No	26	5.85	2.05	

### AR expression in CTCs is negatively correlated with HCC recurrence/progression after hepatectomy

**Figure 2:**
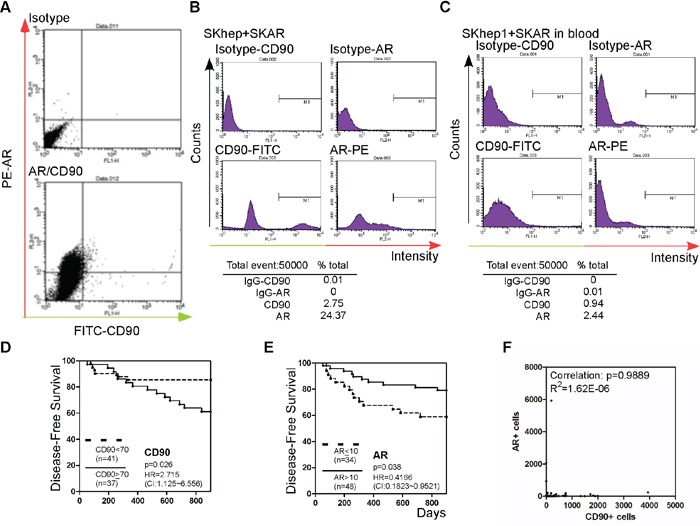
Detection of CTC AR, and CTC CD90+ in patient blood **A.** SKhep1 and SKAR cells were co-stained with CD90 and AR antibodies. Green (x-axis) represents the cells stained with CD90 and IgG-FITC-conjugated secondary antibody, and red (y-axis) represents the cells stained with AR and IgG-PE-conjugated secondary antibody. The upper panel shows isotype (FITC- or PE-conjugated) secondary antibody staining only as the background control. **B.** SKhep1 and SKAR cells were mixed in a 1:1 ratio and then co-stained with CD and AR antibodies. The CD90+ cells represented 2.75% and AR+ cells represented 24.37% of the cell populations. **C.** A SKhep1/SKAR cell mixture (total 10^5^ cells) was added to 10 ml of whole blood, and the PBMCs were then separated. Approximately 20% (v/v) of the PBMCs were co-stained with CD90 and AR antibodies. The CD90+ cells represented 0.94% and AR+ cells represented 2.44% of the PBMC population. **D, E.** The association between HCC disease-free survival (DFS) after hepatectomy and CD90 (D) or AR (E) expression. Patients with a higher number of circulating CD90+ cells (≥70 cells) (n = 37) exhibited poorer DFS than patients with a lower number of circulating CD90+ cells (< 70 cells) (n = 41), whereas patients with a higher number of circulating tumor cells expressing AR (> 10 cells) (n = 48) exhibited better DFS than patients with a lower number of circulating AR+ tumor cells (≤10 cells) (n = 34). **F.** The correlation between AR+ and CD90+ cells was analyzed. The P-value was 0.9889 (R^2^ = 1.62E-06).

Except for the detection of AR and CD90 in primary tumors, we also examined CTCs in the same cohort. Blood samples from 75 patients who had been followed for more than 900 days and passed the quality control check (> 90% of the blood cells were intact) were included in the study. Before measuring patient CTCs, we established the CD90-CTC detection method, which follows Yamashita et al.'s method [21]. We detected the expression of CD90 in SKhep1 cells that had been engineered to stably express AR (high CD90+ cell line [21]; SKAR). We stained the cells with antibodies to AR and CD90, and then subjected them to flow cytometry analysis to detect the expression of the two markers as shown in Figure 2A. The number of CD90+ cells detected was similar to that reported by Yamashita et al. [21], and AR was co-stained in SKAR cells. In order to distinguish AR+ and AR–cells, we mixed SKhep1 parental and SKAR cells and subjected them to CD90/AR co-staining as shown in Figure 2B. In order to mimic the detection of CTCs in blood, we mixed SKAR and SKhep1 cells at a 1:1 ratio and then added 10^5^ cancer cells to whole blood from healthy subjects. Peripheral blood mononuclear cells (PBMCs) were separated from the blood–cancer cell mixture as described in the Materials and Methods section. Approximately 20% (v/v) of the PBMCs were co-stained with AR, CD90, and CD45, and then subjected to flow cytometry analysis as shown in Figure 2C. To exclude the possible detection of PBMC cells, we excluded CD45+ and only counted CD90+ to associate with AR expression. The results showed successful capture of the cancer cells using CD90 and AR staining methods in the blood mixture, and the detection rate was > 90% (Figure 2C, lower panel).

We then examined whether differences in the levels of CD90 and AR expression in CTCs in blood samples from patients who had undergone surgery for HCC (Table 1) correlated with DFS. As shown in Figure 2D, patients with a high number of CTCs expressing CD90 (≥70 cells) had lower DFS than patients with a low number (< 70 cells) of CD90+ cells (p = 0.026). A similar finding was reported by Yang et al. [22]. In addition, we found that patients with a low population of AR-expressing CTCs (≤ 10 cells) had a lower DFS than patients with a high level of AR-positive cells (> 10 cells) (p = 0.038; Figure 2E). Correlation analysis revealed very low no correlation between AR and CD90 expression (R^2^ = 1.62E-06; Figure 2F).

The findings presented in Figures 1 and 2 demonstrate a negative correlation between AR and CD90 expression in both CTCs and primary tumor tissue. The findings also demonstrate that AR expression in CTCs and primary tumor tissue is correlated with postoperative recurrence of HCC, in an opposite pattern.

### AR suppresses CD90 expression partially through DNA methylation and upregulation of histone 3H2A

**Figure 3:**
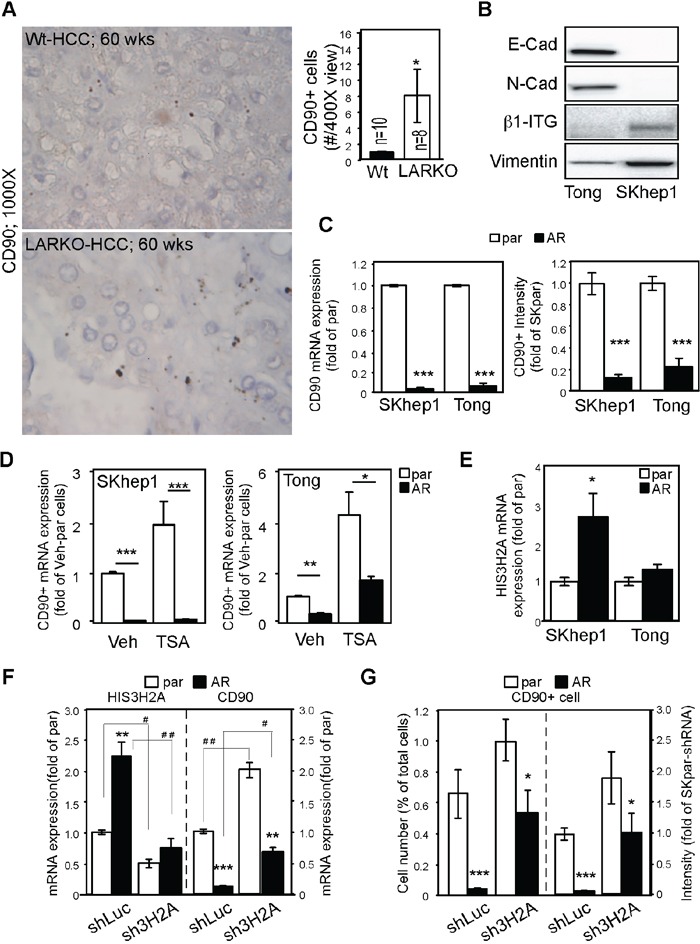
AR suppresses CD90 expression by upregulating Histone 3H2A **A.** Knockout of AR in a carcinogen-induced HCC mouse model enhanced CD90 expression. WT: wild type (upper panels); LARKO: liver-specific AR knockout (lower panels). Photos on the right: Liver tumors from 60-week-old WT or LARKO mice were stained with antibodies to CD90 and observed under a microscope. CD90-positive cells were counted under 400× magnification (left panels). Representative micrographs of intracellular staining of CD90 at 1000× magnification are shown in the right panels. Bar graph on the right: Quantification of CD90+ cells in HCC lesions. **B.** Human mesenchymal-like HCC Tong and SKhep1 cells were detected with various mesenchymal markers, namely E-cadherin (E-Cad), N-cadherin (N-Cad), β1-integrin (β1-ITG), and vimentin. **C.** AR dramatically reduced CD90 mRNA expression (bar graph on the left) and protein expression (bar graph on the right) in SKhep1 and Tong cells. par: parental HCC cells; AR: HCC cells engineered to stably express AR. **D.** TSA treatment partially reversed AR-mediated CD90 suppression in Tong (lane 1 vs. 2, p<0.0001; lane 3 vs 4, p=0.0077), but not SKhep1 cells. (lane 1 vs 2; 3 vs 4, p<0.0001) **E.** AR upregulated Histone 3H2A (HIS3H2A) mRNA expression in SKhep1 (p=0.012) but not Tong HCC cells. **F.** HIS3H2A knockdown reversed the suppressive effect of AR on CD90 mRNA expression. shLuc: short hairpin sequence specific to the luciferase gene; sh3H2A: short hairpin sequence specific to the HIS3H2A gene. HIS3H2A was efficiently knocked down with sh3H2A in both parental HCC cells and AR-SKhep1 cells (left side). The suppressive effect of AR on CD90 expression was partially reversed by sh3H2A (right side). **G.** HIS3H2A knockdown partially restored CD90+ cell numbers (left side). A similar result was seen with CD90 staining intensity (right side). The plotted data were from the mean value of at least three independent experiments, and SD was used to show the variation in the experiments. * Indicates comparisons between par and AR cells, while # indicates comparisons between the knockdown effects of shLuc and sh3H2A. A single * or # represents a significant difference at p < 0.05; a double * or # represents significance at p < 0.01; and a triple * or # indicates significance at p < 0.001.

In order to characterize the relationship between AR and CD90 expression in HCC, we examined their expression levels at various stages of tumor development in a spontaneous HCC mouse model. We found that CD90 was rarely observed in the early (30 weeks) and middle (40 weeks) stages of tumor development (data not shown), but was sporadically detected in the late (60 weeks) stage, which supports the notion that CD90 is a marker of HCC progression [17]. Furthermore, we found that CD90+ cell numbers were detected in the middle (40 weeks; data not shown) and late (60 weeks; Figure 3A, lower panel photos) stages of HCC in liver-specific AR knockout (LARKO-HCC) mice but were rarely detected in wild-type mice with carcinogen-induced HCC (Figure 3A, upper panel photos; and quantitated in Figure 3A, bar graph on the upper right). In order to delineate the molecular mechanism governing the suppressive effect of AR on CD90 expression, we used two different mesenchymal-like (Figure 3B; defined by E-cadherin, N-cadherin, β1-integrin, and vimentin expression) human HCC cell lines (SKhep1 and Tong; CD90-expressing cells) that stably express AR. We found that CD90 mRNA (Figure 3C; bar graph on the left) and protein expression as well as population levels (Figure 3C; bar graph on the right) were dramatically reduced in cells that stably expressed AR. We then tested whether epigenetic modification is involved in AR-mediated CD90 suppression, and found that trichostatin A (TSA) (class I and II histone deacetylase inhibitor) could partially reverse AR-mediated CD90 downregulation in Tong cells (Figure 3D). Additionally, we performed a cDNA microarray experiment on SKhep1 vs. SKAR cells, and found that AR could upregulate histone cluster 3, H2a (HIS3H2A, core histone). We therefore validated HIS3H2A expression in SKhep1 parental vs. AR expressing cells, and found that AR could indeed promote HIS3H2A mRNA expression (Figure 3E). Then, we knocked down HIS3H2A (Figure 3F, left panel) and found a significant reversion of CD90 mRNA in SKhep1 cells (Figure 3F, right panel). Furthermore, knockdown of HIS3H2A also partially reversed AR-mediated CD90+ cell numbers (Figure 3G, left panel) and CD90+ IHC staining intensity (Figure 3G, right panel) in SKhep1 cells. These data are presented in Figure 3 and indicate that AR suppresses the expression of CD90, at least in part, by histone deacetylation and HIS3H2A upregulation.

### AR suppresses HCC cell migration through transcriptome reprogramming

**Figure 4:**
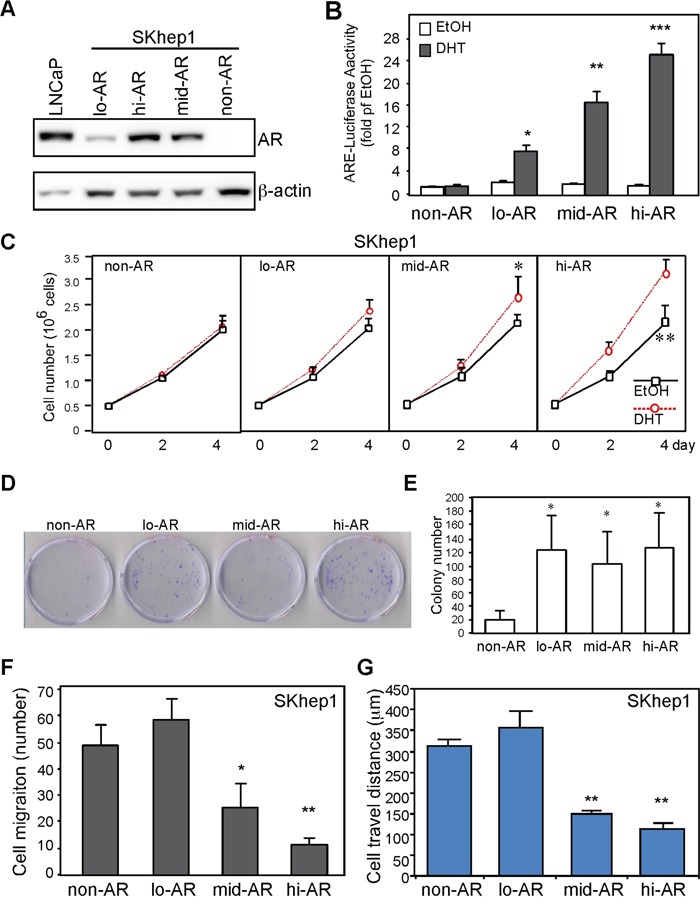
AR expression promotes growth and suppresses HCC cell migration **A.** Establishment of stable clones with differential AR expression. LNCaP: prostate cancer cells expressing AR served as a positive control; lo-AR: SKhep1 cells endogenously expressing low levels of AR; mid-AR: SKhep1 cells engineered to express a moderate level of AR; hi-AR: SKhep1 cells engineered to express high levels of AR. **B.** SKhep1 cells differentially expressing AR were transfected with ARE-luciferase and pRL-TK (transfection control) plasmids to detect AR transactivation. Cells were treated with either ethanol (EtOH) or 10 nM DHT for 24 h before measuring the dual-luciferase activity. All readings were compared with EtOH-treated non-AR cells. **C.** AR promotes short-term cell growth. The non-AR, lo-AR, mid-AR (p=0.039), and hi-AR (p=0.006) cells were seeded (5 × 10^5^ cells/dish) and cultured for 4 days. Cell numbers were counted and plotted. **D, E.** AR promotes long-term cell growth. The non-AR, lo-AR, mid-AR, and hi-AR cells were seeded (10^3^ cells/dish), cultured for 14 days, and then stained with trypan blue. “D” is the representative image to demonstrate AR effect on colony size. “E” is the statistic result of colony number. **F, G.** AR suppressed cell migration (F; Boyden chamber transwell cell migration assay) and travel distance (G; real-time cell mobility assays) in an expression-dependent manner. Compared to non-AR cells, cell migration number and travel distance were comparable in lo-AR cells but were markedly lower in mid-AR and hi-AR cells. The plotted data were from the mean value of at least three independent experiments, and SD was used to show the variation in the experiments.

We also investigated the effects that AR has on cancer cell migration. We established three SKAR clones with differential expression of AR (Figure 4A; SKAR5 > SKAR12 > SKAR4 > SKhep1 → hi- (high) > mid- > lo- (low) > non-detectable AR expression), and found that AR transactivation activities were positively correlated with AR expression levels (Figure 4B). We found that AR promoted short-term (4 days; Figure 4C) and long-term (2 weeks; Figure 4D, 4E) HCC cell growth regardless of AR expression level, which is consistent with a previous finding [3]. However, AR suppressed cell migration (Figure 4F; Boyden chamber assay) and migratory distance (Figure 4G; real-time microscopic observation) in an expression level–dependent manner. These results indicate that AR promotes cell growth and suppresses cell mobility in the same cells.

**Figure 5:**
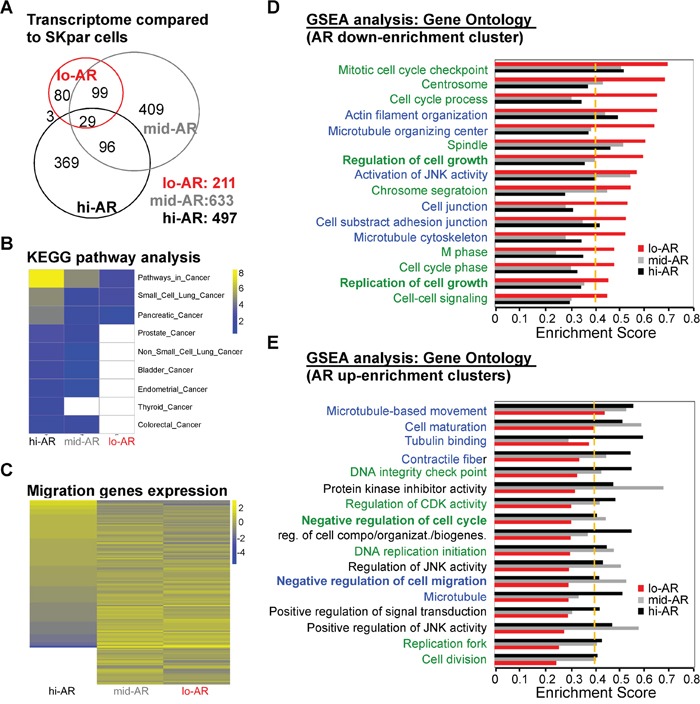
Systems biological analysis revealed that AR-mediated reprogramming of the transcriptome suppressed the expression of migration-related gene clusters **A.** We used cDNA microarray analysis to compare the number of genes expressed in lo- (211 genes), mid- (633 genes), and hi-AR (497 genes) cells with that in non-AR cells. The numbers of overlapping genes were: lo-AR vs. mid-AR = 128 genes; mid-AR vs. hi-AR = 125 genes; lo-AR vs. hi-AR = 32 genes. The number of overlapping genes in all three cell types was 29. **B.** KEGG pathway analysis of microarray data in lo-AR, mid-AR, and hi-AR cells revealed a positive correlation between AR expression and the expression of cancer-related genes. **C.** The Gene Ontology analysis of the migration-related genes in lo-AR, mid-AR, and hi-AR cells revealed an AR-dependent ascending or descending pattern of gene expression. **D, E.** Gene set enrichment analysis (GSEA) was used to study the effect of AR on gene expression. The green-colored label represents the gene clusters associated with cell cycle, while the blue-colored label represents cell mobility–associated clusters. The ratio of cell cycle to migration-related genes was 10:6 in the AR down-enrichment clusters and 6:7 in the AR up-enrichment clusters. A change of > 0.1 in the Enrichment Score (ES) and an ES threshold > 0.4 were considered as significant changes in the gene clusters. UP: upregulation; DN: downregulation; KD: knockdown; KO: knockout.

In order to understand how AR suppresses the mobility of HCC cells on a global scale, we used a cDNA microarray to observe the effect of AR on the transcriptome (Figure 5). We found a gene expression shift between lo-AR cells and mid-AR cells and between mid-AR cells and hi-AR cells. There was an overlap of 128 genes between the lo-AR cells and the mid-AR cells (~20% of mid-AR altered genes), and an overlap of 125 genes between the mid-AR cells and the hi-AR cells (~25% of hi-AR altered genes). However, when the expression profiles were compared between lo-AR and hi-AR cells, only 32 genes overlapped (~6% of hi-AR altered genes) (Figure 5A).

In order to understand the transcriptome shift seen in cells with differential AR expression, we performed pathway analysis using the Kyoto Encyclopedia of Genes and Genomes (KEGG) platform and Gene Ontology database. KEGG analysis of the microarray data revealed that the levels of cancer-related pathways increased in an AR expression–dependent manner (Figure 5B). However, we found that expression of migration-related genes was dependent on the level of AR expression (Figure 4C). We then used gene set enrichment analysis (GSEA) to better understand this discrepancy (ontology enrichment analysis; GSEA: C6) (Figure 5D, 5E). Several of the AR-dependent down-enrichment clusters were related to cell migration clusters (Figure 5D, blue text). On the contrary, several AR-dependent up-enrichment clusters were related to negative regulation of cell migration (Figure 5E).

### AR promotes anoikis of circulating tumor cells by facilitating amorphosis

**Figure 6:**
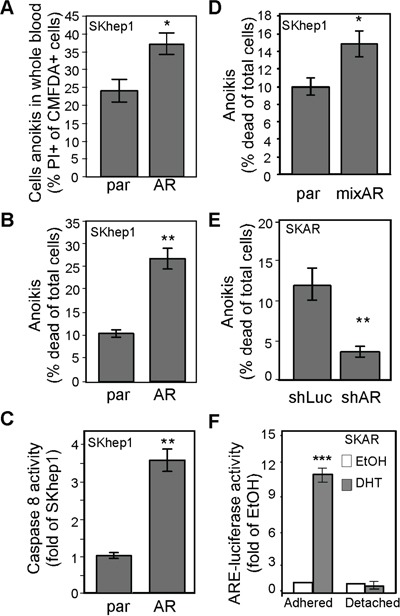
AR-mediated anoikis of CTCs might be due to cytoskeletal rearrangement **A.** AR promoted HCC cell anoikis in whole blood. The parental (par) and SKhep1 cells (AR) were pre-incubated with CMFDA, mixed with whole blood, and then further incubated in non-adhesion conditions to measure cell anoikis. The percentage of CMFDA double-positive cells was higher in AR cells than in par cells. **B.** AR promoted anoikis in regular culture medium. Cells were incubated in DMEM in adhesion conditions, and cell death was measured with PI (propidium iodine; 5 μM). The percentage of cells exhibiting anoikis was higher in AR cells than in par cells. **C.** AR overexpression resulted in increased Caspase 8 activity in non-adhesion cells. **D.** Anoikis was promoted in mixed AR stable clones but not in parental cells. **E.** AR knockdown resulted in reduced anoikis. SKAR cells were infected with AR-specific (shAR) or luciferase-specific (shLuc) shRNA. Anoikis was detected as described above. The percentage of cells exhibiting anoikis was lower in shAR-infected cells than in shLuc-infected cells. **F.** ARE transactivation activity was abolished in non-adhesion conditions. The SKAR cells were co-transfected with ARE-luciferase and pRL-TK (transfection control) constructs, treated with EtOH or 10 nM DHT, and then analyzed for dual-luciferase activity in either adhesion (p<0.0001) or non-adhesion conditions. ARE-luciferase activity dramatically increased in DHT-treated cells in adhesion conditions but not in non-adhesion conditions. The plotted data were from the mean value of at least three independent experiments, and SD was used to show the variation in the experiments. A single * label represents significance at p < 0.05, and a double * label represents significance at p < 0.01.

Studies have shown that the number of CTCs is positively correlated with the risk of cancer recurrence. We therefore tested whether AR facilitates anoikis of circulating HCC cells and found that the degree of anoikis of HCC cells in whole blood (Figure 6A) and in culture medium (Figure 6B) was influenced by the degree of AR expression. We also found that AR-promoted anoikis is a Caspase 8-related event (Figure 6C). The AR effect on anoikis was not only observed in single clones that stably express AR but also in mixed clones (Figure 6D), which excluded the single clone effect. In addition, AR-mediated anoikis was abolished in AR knockdown cells (Figure 6E). The results indicate that AR is a specific promoter of anoikis in HCC cells. We measured AR transactivation activity in HCC cells, and found that it was absent in detached cells (Figure 6F), which indicates that AR-mediated anoikis might not be a transcription-related event.

**Figure 7:**
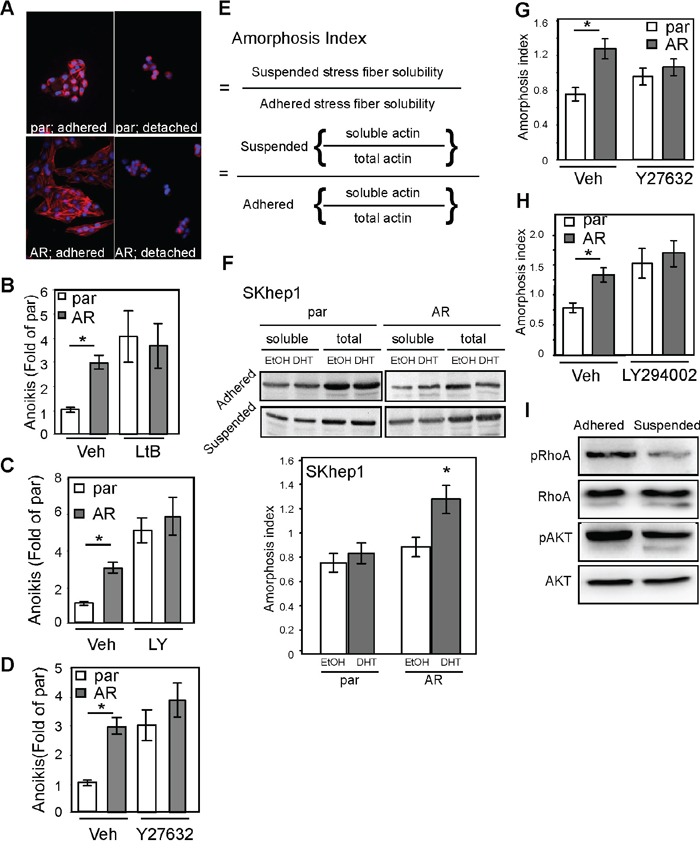
AR promotes anoikis by enhancing cellular amorphosis **A.** AR-expressing SKhep1 cells exhibited higher stress fiber–dissolving capacity than par cells. The cells were treated with EtOH or DHT, maintained in adherent or detached conditions for 24 h, and then fixed and co-stained with rhodamine phalloidin and DAPI. Red fluorescence represents actin stress fibers and blue represents cell nuclei stained by DAPI. **B.** Latrunculin B1 (LtB) abolished AR-mediated anoikis. The degree of anoikis was compared among SKhep1, par, and AR (p=0.02) cells with or without pre-treatment for 3 h with 0.5 μM LtB. The percentage of cells exhibiting anoikis was compared between par and Veh-treated cells. **C, D.** PI3K/AKT inhibitor LY294002 (LY; C) and ROCK inhibitor (ROCK inh.; D) reduced the degree of AR-mediated anoikis. The degree of anoikis was compared among SKhep1, par, and AR cells with or without co-treatment with LY/ROCK. The percentage of cells exhibiting anoikis was compared between par and Veh-treated cells. All experiments were performed at least three times, and the data represent the mean values from three independent experiments. **E.** The amorphosis index is a measure of solubility of actin stress fibers in detached conditions divided by that in attached conditions. The solubility of α-actin stress fibers was determined by dividing the amount of soluble actin by the total amount of actin. **F.** Upper image: The par and AR cells were treated with EtOH or 10 nM DHT; soluble α-actin (cell lysate with PBS) and total actin (cell lysate with 1 μM NP-40 RIPA buffer) were then measured using an immunoblot assay with α-actin antibody. The amorphosis index was higher in DHT-treated AR cells. Lower bar graph: The optical density of the immunoblots was compared between par and AR SKhep1 cells using the equation presented in (E). The readings were compared between DHT- and EtOH-treated cells. **G–H.** LY (G) and ROCK (H) inhibitor abolished AR-promoted (p=0.031) cell amorphosis in DHT-treated cells. **I.** The degree of phosphorylation of Rho A (pRhoA) and AKT (pAKT) was significantly lower in SKAR cells in detached conditions than in SKAR cells in attached conditions. The plotted data were from the mean value of at least three independent experiments, and SD was used to show the variation in the experiments. A single * label represents significance at p < 0.05, and a double * label represents significance at p < 0.01.

The cell anoikis mechanism could be related to cytoskeletal rearrangement [23, 24] or cell adhesion [25, 26]. We found that AR could alter the cytoskeleton when the actin stress fibers were dissolved in detached conditions (Figure 7A), which suggests that cytoskeletal rearrangement might be involved in AR-mediated anoikis. To better understand that finding, we exposed cells to latrunculin B1 (LtB1), an actin fiber chelator, and found that the degree of anoikis was diminished (Figure 7B). In addition, incubation of cells in the presence of inhibitors of actin rearrangement, LY294002, PI3K/AKT blocker [27] (Figure 7C), and Y-27632, ROCK inhibitor [28] (Figure 7D), also reduced the degree of AR-mediated anoikis. These data implicating actin cytoskeletal rearrangement might be involved in AR-mediated anoikis.

Evidence has been put forward that amorphosis, a type of apoptosis induced by the disruption of cytoskeletal rearrangement, leading to a distorted cell shape, is one of the mechanisms governing anoikis [29]. In this study, we developed a method to quantify the degree of amorphosis (Figure 7E; Materials and Methods section). By quantifying the immunoblot result of α-actin (Figure 7F image) from cell extracts, we found that AR promoted amorphosis (Figure 7F bar graph). We then tested whether AR-mediated amorphosis could be blocked by LY294002 or Y27632. Indeed, AR-induced amorphosis was blocked by these two signal inhibitors (Figure 7G, 7H). Finally, we measured RhoA (ROCK substrate) and AKT (PI3K substrate) phosphorylation and found that the levels of pRho and pAKT were lower in detached cells than in attached cells (Figure 7I).

The data presented in Figures 6 and 7 suggest that AR overexpression promotes anoikis of CTCs by inducing cellular amorphosis.

## DISCUSSION

### Tumor heterogeneity and suppression of AR-mediated HCC recurrence

HCC patients at Barcelona Clinic Liver Cancer (BCLC) stage B (early cancer development) or stage C (intermediate cancer development) disease are normally eligible to receive hepatectomy [30, 31]. Theoretically, the cellular characteristics of hepatectomy patients are considered in the early developmental stages of cancer, but can exhibit characteristics that have been shown to be associated with recurrence, which implies that tumor heterogeneity exists even in the early stages of cancer [32]. Prognostic factors such as tumor size, TNM stage, and AFP levels are not sufficient predictors of HCC recurrence after hepatectomy [19]. Colonization of surviving tumor cells in the circulation is one hypothesis for HCC recurrence [16]. Although particular subpopulations of CTC CSPC might serve as a marker of HCC recurrence, the regulatory mechanisms are poorly understood.

**Figure 8:**
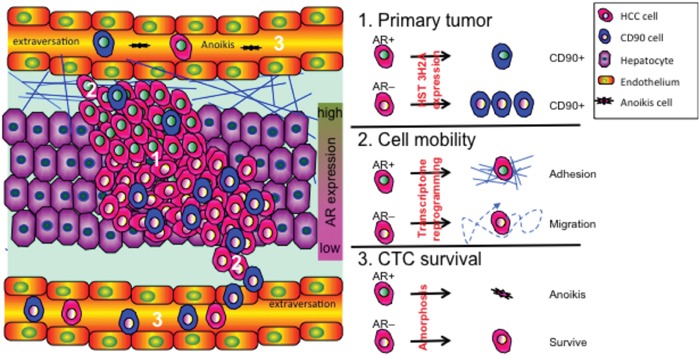
AR expression reduces the risk of HCC recurrence via a three-pronged mechanism The three major mechanisms governing cancer recurrence are as follows: 1. CD90 expression in primary tumors increases the risk of cancer cell colonization; 2. High HCC cell mobility increases the risk of cancer cell extravasation into vessels; and 3. High rates of CTC survival in the circulation increase the risk of CTCs returning to the liver. On the molecular level, overexpression of AR suppresses the recurrence of HCC by: (1) interacting with Histone 3H2A, which results in decreased expression of CD90 in primary tumor cells, (2) suppressing cell mobility through a transcriptome shift that facilitates cell adhesion, and therefore cell movement, and (3) facilitating anoikis of CTCs by enhancing cellular amorphosis.

AR has been reported to suppress HCC metastasis [2, 11]. Boix et al. [20] using receptor binding assay (RIA) conducted in 1995 were the first paper to associate AR expression in HCC disease progression. There is hindered information in that paper. 1. In general, this field accepted the idea that AR promotes HCC development. However, Boix found AR is failed to associate with poor progression outcome. At that time, Boix cannot explain this phenomenon. 2. The criteria for HCC patient undergo hepatectomy surgery is quite controversial in 1995. The BCLC for liver surgery has confirmed the criteria after 2000. [30] Therefore, the patient inclusion criteria in Boix's paper might be inconsistent with current standard procedure. 3. The method of AR measurement was RIA in Boix's paper, which is not a very specific AR detection method from now-a-day antibody technology point of view. Re-evaluation of AR expression in HCC disease progression is no doubt an important work for this issue. In this study, we provide evidence for a three-pronged mechanism by which AR protects against HCC recurrence (Figure 8). First, AR suppresses CD90 expression in primary tumors partially by upregulating HIS3H2A, which consequently results in a reduction in CD90+ tumor cell populations in the blood, thereby reducing the risk of recurrence. This mechanism explains the negative association between the expression of AR and that of CD90 in CTCs and also explains the negative association between AR expression and DFS of patients after hepatectomy.

Our finding that AR mediates the suppression of CD90 expression implies not only that AR is a potential biomarker of disease recurrence but also that AR could be a target for gene therapy, possibly using the CRISPR/Cas9-activator (clustered, regularly interspaced, short palindromic repeats/CRISPR-associated protein) genome editing system [33, 34]. For example, in clinical practice, some patients in the advanced stages of HCC, such as BCLC stage C or unresectable HCC, are advised to undergo hepatectomy after transarterial chemoembolization or other downstage interventions have failed to control cancer progression [35]. In these patients, AR-targeted therapy performed during downstaging interventions might increase the effectiveness of chemo- or radioembolization, thereby sparing the need for hepatectomy or liver transplantation.

### Novel and translational roles of AR expression in HCC recurrence

Colonization of CTC CSPC (e.g., CD90) only partially explains the processes of the recurrence of HCC and other heterogeneous diseases. The second mechanism through which AR protects against HCC recurrence is its ability to suppress cell mobility at the transcriptome level. To the best of our knowledge, this is the first study to describe such a phenomenon in HCC.

Although the presence of CTCs expressing CSPC markers partially explains the high rate of recurrence after hepatectomy, the number of CTCs in general is also responsible for HCC recurrence [36]. Therefore, the third mechanism through which AR protects against HCC recurrence is its ability to suppress the survival of CTCs by inducing the disruption of cytoskeletal rearrangement. This process does not involve the transactivation function of AR (Figure 6F) but instead is regulated by non-genomic signaling processes. We found that this AR non-genomic anoikis–amorphosis promotion effect might be governed by the PI3K/AKT and ROCK signaling pathways. Studies have shown that the transient activation of AKT by AR is related to cell growth and resistance to apoptosis in prostate cancer cells [37]. Although AR-AKT signaling is common in prostate and liver cancers, the cellular outcome differs between the two diseases. The difference in outcome might be due to different conditions (attached vs. detached → anti-apoptosis vs. anoikis) that reflect different types of disease progression (primary tumor vs. CTC → tumor promoter vs. recurrence suppressor).

In this study, we have described three mechanisms through which AR suppresses HCC recurrence. Our results indicate that enhancing the expression of AR in CTCs might reduce the risk of HCC recurrence.

## MATERIALS AND METHODS

### Study subjects

In order to examine the association between AR expression and cancer recurrence, we prospectively enrolled patients who had undergone hepatectomy at the Organ Transplantation Center, China Medical University Hospital (CMUH), Taiwan, during the period 2010–2012. Primary HCC tumor specimens and 20 ml of whole blood were collected with informed consent from 110 patients, of whom 97 patients had been followed for up to 900 days. Of the blood samples collected, 87 patient samples passed the quality control check and were included in the CTC analyses. All paraffin-embedded tumor tissue blocks from the 110 patients were subjected to IHC studies. Detailed patient demographic data are presented in Supplementary Figure 1. This study was approved by the Institutional Review Board of the CMUH (IRB# DMR100-IRB-088).

### Measurement of AR and CD90 expression in CTCs

PBMCs from patients were used to detect the expression/positivity of AR and CD90 in CTCs. In brief, 10 ml of whole blood and 5 μM EDTA were mixed with FICO buffer (GE Healthcare Ficoll-Paque™PLUS, 17-144-03; blood: Ficoll = 5:3; v/v) and then centrifuged to isolate the PBMCs. The CTCs were first defined by size; then the lymphocyte were removed by excluding CD45+ cells. The cells were then fixed with cold methanol (−20°C) and then stored at −80°C. For the detection of CTCs, PBMCs (200 μl) were washed and then incubated with CD90 and AR antibodies in the presence of 0.5% NB-40 (Sigma) for 1.5 h. Cells were then incubated with PE- or FITC-conjugated 2**°** antibody IgG for 30 min and analyzed by flow cytometry (FACS, BD FACAria).

### Amorphosis index

Since the definition of amorphosis is the change in solubility of cytoskeletal proteins between the attached and detached states, we measured cellular stress fiber solubility with different stringencies of lysis buffer, and detected α-actin (Santa Cruz, CA) using an immunoblot assay. The amorphosis values were then quantified from densitometric data from the blots using BioRad QuantDot (BioRad) as shown in Figure 7E. Soluble α-actin was isolated by adding double-distilled water to the cells (10^5^ cells/200 μl H_2_O), aspirating the mixture 30 times with a syringe (#30 needle), and centrifuging it at 1000 × g for 10 min. The insoluble fraction was then discarded. Total α-actin was isolated by adding lysis buffer (containing 0.05% NB-40; 10^5^ cells/200 μl buffer) to the cells, aspirating the mixture 30 times with a pipette (200 μl tip), and centrifuging it at 1000 × g for 10 min. The insoluble fraction was then discarded.

### Cell culture, chemicals, plasmids, lentiviral-based gene transduction, and stable expression of cell lines

Cells were maintained in DMEM with 10% FCS (Invitrogen), 1% L-glutamine, and 1% penicillin/streptomycin as described previously [38]. The cell lines HEK293T and SKhep1 (ATCC; HTB52) were purchased from ATCC, and Tong [39] cells were provided by YS Jou (Academia Sinica, Taiwan). HCC clones were engineered to stably express AR by transfection of pBabe-human AR cDNA into SKhep1 or Tong cells and then selected after exposure to puromycin (10 μM) for the indicated periods of time. Single colony SKAR4 (lo-AR), SKAR12 (mid-AR), SKAR5 (hi-AR; SKAR), and Tong-AR cells were used in this study.

The following antibodies and chemicals were used: CD90, E-cadherin, phospho-RhoA, AKT, phospho-AKT (Cell Signaling Technology); RhoA, AR (N-20), vimentin, integrin β1, and actin (Santa Cruz); propidium iodine (Sigma-Aldrich; 5 μM); DHT (5α-dihydrotestosterone; PerkinElmer); flutamide (1 μM, PerkinElmer); FAK inhibitor (1,1,2,4,5-benzene-tetramine; NSC227698; Merck; 1 μM); Src inhibitor (4-(4′-phenoxyanilino)-6,7-dimethoxyquinazoline; Merck; 50 nM); Akt inhibitor (2-(4-morpholinyl)-8-phenyl-1(4H)-benzopyran-4-one hydrochloride; LY294002; Sigma-Aldrich; 1.5 μM); ROCK1/2 inhibitor ((R)-(+)-trans-N-(4-pyridyl)-4-(1-aminoethyl)-cyclohexanecarboxamide; Y27632; Merck; 150 nM); Latrunculin B (Sigma-Aldrich; 1 μM); Epz (10 μM; EPZ6438; MedChem Express); suberanilohydroxamic acid (5 μM; Sigma-Aldrich); TSA (1 μM; Sigma-Aldrich); 5′-aza-2′-deoxycytidine (5 μM; Sigma-Aldrich); CMFDA (5-chloromethylfluorescein diacetate; Invitrogen, CellTracker).

### Lentiviral-based gene delivery

shRNAs 1. shRNA toward β1-integrin and scramble siRNA were used as reported previously [10, 11]. The pLKO-shLuciferase and shHIST3H2A (TRCN0000106910) plasmids were obtained from the National RNAi Core Facility Platform (Institute of Molecular Biology/Genome Research Center, Academia Sinica, supported by the National Core Facility Program for Biotechnology; grant NSC100-2319-B-001-002). The pBabe and pWPI (Addgene) vector-based AR cDNA expression plasmids were constructed as previously reported [3]. The lentiviral production and infection procedures used in this study followed those reported previously [11]. In brief, psPAX2 (packaging plasmid) and pMD2G (envelope plasmid) (Addgene) were co-transfected into HEK293T cells. We then harvested virus-containing media to infect the HCC cells. The GFP+ cell populations, as determined by flow cytometry analysis (BD LSR II Flow Cytometry), were used to test the infection efficiencies.

### Immunohistochemistry, fluorescence microscopy, and quantitation of staining

In general, the histological studies were performed as described in previous studies, with slight modification [40, 41]. For general histologic inspection, we treated tissue sections (2 μM) with hematoxylin and eosin, or stained sections with antibodies specific for AR and CD90 with an ABC kit (Vector Laboratories) to enhance the staining signals. Staining intensity was scored according to the Allred scoring system as described previously [42, 43]; the reference values are shown in Supplementary Figure 1. The proportion of cells that stained positive for AR or CD90 was graded using a five-point scale according to the proportion of positive cancer cells (1: < 1/100; 2: 1/100 to 1/10; 3: 1/10 to 1/3; 4: 1/3 to 2/3; and 5: > 2/3). The intensity of staining was also graded on a five-point scale as follows: 1: none; 2: weak; 3: intermediate; 4: mid-strong; 5: strong. The proportions and intensity scores were then added together and compared with clinical data. The slides were independently examined by two coauthors (H-C Lai, MD; CC Yeh, MD) who were blinded to the clinicopathologic data. When there was a discrepancy (score difference > 2) between samples, both pathologists reassessed the slides using a double-headed microscope, and a consensus was reached. Finally, associations between the scores and the clinical data were investigated by another coauthor (LB Jeng, MD).

### Maintenance of animals and generation of carcinogen- and HBV-related HCC mouse models

All of the animal experiments followed the Guidelines for the Care and Use of Laboratory Animals (Ministry Of Sciences and Technology, Taiwan) and were approved by the China Medical University Committee of Laboratory Animal Welfare (101-38-N). Liver-specific ARKO (LARKO) and HBVtg-LARKO mice as well as the spontaneous HCC mouse model were generated using previously described protocols [3, 4].

### Gene expression assays

#### Immunoblot assay

AR expression was analyzed using a previously described immunoblotting assay [44, 45]. Briefly, protein extracts were subjected to SDS-PAGE and then transferred to a PVDF membrane (BioRad). The membrane was then incubated with the appropriate antibodies. After incubation, ECL reagent (BioRad) was applied to the membrane, and the signals were detected by Chemidoc XRS+ (BioRad) with a charge-coupled device (CCD) camera.

#### Luciferase promoter assay

The assay was performed as previously described [46, 47]. Briefly, pGL3-ARE (androgen response element–driven luciferase reporter plasmid) and pRL-TK (thymidine kinase promoter–driven Renilla luciferase plasmid) were co-transfected into cells. The cells were then treated, lysed (Promega, WI, USA), and subjected to a luminescence reader (Promega).

#### Real Time RT-PCR of mRNA expression

The RNAs were extracted from cells or tissue as previously described [48–50]. The primers for CD90 were: Forward: 5′-CTAGTGGACCAGAGCCTTCG; Reverse: 5′-TGCAGTGCACACGTGTAGGT. The primers for HIST3H2A were: Forward: 5′-ACAAGGCCAAGGGCAAGT; Reverse: 5′-TCTGAAAAGAGCCTTTATGTCCA.

### Fluorescence microscopy for cytology study

Cell morphology was studied by staining for α-actin, stress fibers, and nuclei. Cells were seeded on four-chamber glass culture slides and then fixed with 4% paraformaldehyde (Sigma-Aldrich) in PBS. After washing with PBS, rhodamine phalloidin, a high-affinity α-actin probe conjugated to the red–orange fluorescent dye tetramethylrhodamine (Sigma-Aldrich; 1 μM), was mixed with DAPI (Sigma-Aldrich; 10 nM) and then applied to cells for 5 min. The cells were then sealed with cover slides and observed under a fluorescence microscope (ECLIPSE 80i; Nikon). Images were recorded using a CCD camera (DS-Qi1Mc) and then analyzed with imaging software (NIS-Elements BR; version 3.1).

### Microarray and systems biological analysis

Total RNA was extracted and then reverse transcribed to generate cDNA, which was then subjected to microarray analyses as previously described [51, 52], with slight modifications. Fluorescence-labeled cDNA was generated from 20 mg of RNA with oligo-dT primer (New England Biolabs, Inc.) and Superscript II (Life Technologies, Inc.) in the presence of labeled nucleotides (Amersham Pharmacia Biotech). The fluorescence-labeled cDNA was then pooled and purified (Millipore). The probe and cDNA were incubated on a microarray chip (Corning, NY), washed, dried, and then scanned with a GMS418 scanner (Affymetrix, CA). After scanning, the imaging data were analyzed with GenePix Pro software (Axon Instruments, CA) [53]. The significance of changes in the expression ratios of the transcript were calculated using Significance Analysis of Microarrays software [54]. All results were obtained from two independent sets of experiments, and the readings were the mean values of reproducible genes from each set of experiments. The readings of the microarray data were compared within controls (0 nM DHT treatment; or non-AR cells) and experimental groups (1 or 10 nM; or low-, mid-, hi-AR cells). A fold change > 1.5 was used as the threshold.

The microarray data were analyzed using a series of systems biology approaches. We subjected microarray data to GSEA (C5 and C6 gene sets; http://www.broadinstitute.org/gsea/msigdb/index.jsp), KEGG (http://www.genome.jp/kegg/kegg3a.html), and MetaCore pathway analyses (http://thomsonreuters.com/metacore/) with the support of the Bioinformatics Center at National Cheng-Kung University, Taiwan (the TMBD Bioinformatics Core [http://www.tbi.org.tw], funded by National Core Facility Program for Biotechnology, MOST 104-2319-B-400-002). A change of > 0.1 in Enrichment Score (ES) and an ES threshold > 0.4 were used as the selection criteria for the GSEA data (Figure 5).

### Cell migration, colony growth, anoikis, and amorphosis assays

#### Cell migration assay

The cell migration assay was performed as previously described [55]. Briefly, one thousand cells were seeded onto the upper chamber of 8-μM pore transwell plates (Invitrogen). The lower chamber was filled with 20% FCS, and then the cells were incubated at 37°C overnight to allow cell migration. Then, the inner part of the upper chamber was scraped out with a cotton swab, and then the well was stained with trypan blue. The cell numbers were counted under a 100× microscope.

#### Mobility assay

The cell mobility assay was performed as previously described [56], with slight modifications. Briefly, cells (1.5 × 10^5^/35-mm dish) were observed and photographed every 15 min for 8 h using a Time-Lapse microscope (NanoEnTek, Korea). The series of images was stacked by ImageJ software (NIH; http://imagej.nih.gov/ij/index.html) and saved in AVI film format. The cell travel distance was recorded by the track function of ImageJ, calculated, and imported to an Excel file for interpretation.

The colony formation assay was performed as previously described [41, 57]. Briefly, 1000 cells were seeded onto 60-mm dishes and cultured for 2 weeks. Cells were then fixed in 4% formalin and then stained with crystal violet (0.05%). Images of colony formation were photographed and quantified by ImageJ software.

### Statistics and disease-free survival

Each in vitro assay was performed in triplicate, and data were derived from at least three independent sets of experiments. Values were calculated and compared using Student's t-test. DFS was calculated using Kaplan–Meier survival curves and the log-rank test. In all analyses, a P-value < 0.05 was considered to represent statistical significance.

## SUPPLEMENTARY FIGURE


