# Supplementary material for: Androgen receptor mitigates postoperative disease progression of hepatocellular carcinoma by suppressing CD90+ populations and cell migration and by promoting anoikis in circulating tumor cells
Source: Oncotarget. 2016 Jun 20;7(29):46448–65. doi: 10.18632/oncotarget.10186 (PMC5216809; doi:10.18632/oncotarget.10186)
Supplement: Supplementary file 1 [file oncotarget-07-46448-s001.pdf]

Androgen receptor mitigates postoperative disease progression of hepatocellular carcinoma by suppressing CD90+ populations and cell migration and by promoting anoikis in circulating tumor cells

SUPPLEMENTARY FIGURE

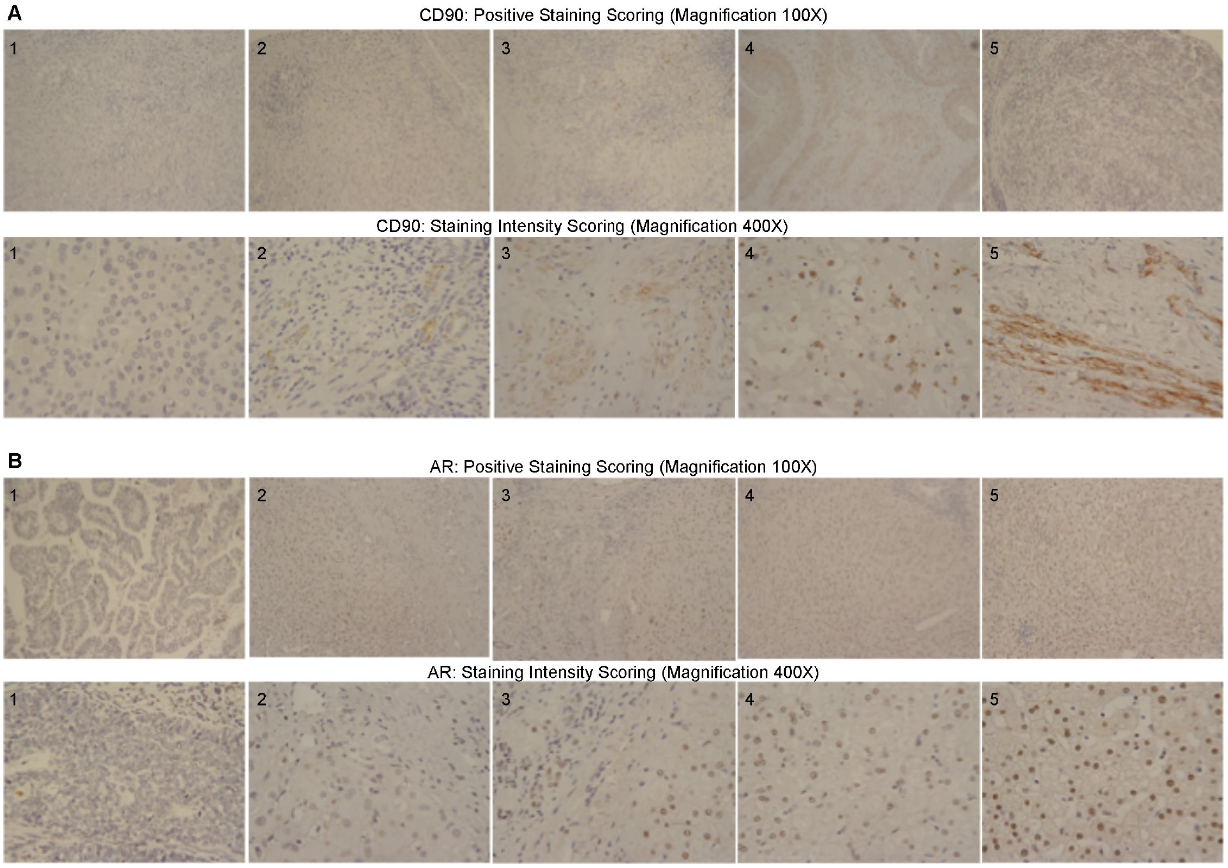

Supplementary Figure S1: Scoring of immunohistochemical staining of CD90 and AR. A. CD90 staining scores in the HCC patient cohort. Upper panels demonstrate low to high positivity observed under 100X magnification and lower panels show low to high intensity staining as observed under 400X magnification. **B.** AR staining scores in the HCC patient cohort. Upper panels represent staining positivity at 100X magnification and lower panels show staining intensity at 400X magnification.
